# Managerial Myopia and Firm Green Innovation: Based on Text Analysis and Machine Learning

**DOI:** 10.3389/fpsyg.2022.911335

**Published:** 2022-06-20

**Authors:** Xin Liu

**Affiliations:** School of Business, Renmin University of China, Beijing, China

**Keywords:** managerial myopia, green innovation, upper echelons theory, text analysis, equity incentives, institutional investors

## Abstract

Green innovation plays an important role in reducing pollution and promoting sustainable development. However, not all managers, as decision-makers of enterprises, have a long-term vision to implement green innovation. The impact of managerial myopia on firm green innovation has not been examined by existing literature. Drawn on time-oriented theory in social psychology and upper echelon theory in management, this paper puts forward the relationship between managerial myopia and firm green innovation, and then constructs a managerial myopia index by text analysis and machine learning. Using a sample of publicly listed Chinese firms from 2009 to 2020, this paper finds that managerial myopia is significantly negatively associated with firms’ green innovation. Every one standard deviation increase in managerial myopia makes enterprise green innovation decrease by about 1.9% standard deviation. Further analysis reveals that equity incentives and institutional investors both weaken the negative effect of managerial myopia on green innovation. This study contributes to the literature on green innovation and upper echelons theory and it also has several implications for firms’ engagement in green innovation.

## Introduction

In the recent decade, the global community has shown serious concerns regarding the deterioration of the natural environment, the rising global temperatures, and the extinction of some species. In the long run, the effective means to reduce environmental degradation and achieve sustainable development mainly rely on green technological innovation (Aragaón-Correa et al., 2008; Acemoglu et al., 2012; Mensah et al., 2018).

In 2020, China released 10.67 billion metric tons of carbon dioxide emissions, ranking first in the world.^1^ As a result, research on Chinese green innovation is particularly important and intriguing. As a developing country with the largest carbon emissions, China has been endeavoring to facilitate sustainable development together with the other major economies. China is committed to achieving green development and has set the goal of achieving a carbon peak by 2030 and carbon neutrality by 2060. Green innovation enables countries to produce energy-saving products and apply carbon-free technologies (Garrone and Grilli, 2010), and finally achieve sustainable transformation of economic structure (Alvarez-Herránz et al., 2017). As a new sustainable development pattern, green innovation has been highly valued by the Chinese government and enterprises. Thus, studying green innovation in the context of China has become more and more important, and it attracts more attention from managers and researchers (Yuan and Xiang, 2018; Zhang et al., 2020; Huang et al., 2021; Ren et al., 2021; Xu et al., 2021; Zhou M. et al., 2021).

Green innovation can not only benefit firms by saving energy and cost, but also improve the ecological environment quality by reducing emissions for sustainable development (Sierzchula and Nemet, 2015; Dangelico et al., 2017).

Although green innovation can increase a firm’s sustainability, it has the characteristics of high R&D expense and a high risk of failure (Holmstrom, 1989). However, not all managers, as decision-makers of enterprises, have a long-term vision to implement green innovation. Upper echelons theory suggests that executives’ experiences, values, cognitions, and personalities will affect firms’ strategy choices and outcomes (Hambrick and Mason, 1984; Hambrick, 2007). As firm’s decision-maker, executives have the power to influence green innovation strategy, thus how executives’ personal traits affect enterprises’ engagement in green innovation is important to be explored. In this study, we explore the effect of an important and interesting personal trait on green innovation, that is, managerial myopia.

Managerial myopia, which originated from the time orientation theory in social psychology, means that managers’ time cognition is short-term oriented and they are subjectively focused on and value the present (Stein, 1989; Laverty, 1996; Lin et al., 2019). Instead of focusing on the future development of the enterprise, myopic managers are more inclined to focus on the benefits that can be satisfied immediately. This short-term time orientation is generally regarded as an innate and stable personal trait (Goldrich, 1967) and a subconscious process (Zimbardo and Boyd, 1999). It is worth exploring the impact of managerial myopia on firm green innovation and the regulatory effect of what factors will regulate the relationship between them.

The management discussion and analysis (MD & A) disclosed in the annual report of listed companies is the manager’s review of the enterprise’s operation status and the prospect of future development. It is widely recognized by researchers to capture the subconscious cognition and characteristics of managers (Li, 2010; Loughran and McDonald, 2011). Therefore, this paper takes the management discussion and analysis (MD & A) in the annual reports of Chinese A-share listed companies as the object, determines the Chinese “short-term orientation” word set through text analysis and machine learning methods, and then constructs the managerial myopia index by dictionary method. Using a sample of publicly listed Chinese firms from 2009 to 2020, this paper finds that managerial myopia has a negative effect on firms’ green innovation. Every one standard deviation increase in managerial myopia makes enterprise green innovation decrease by about 1.9% standard deviation. Such effect remains significant after a series of robustness checks including alternative sample interval, alternative dependent variable measures, alternative model regression, and endogenous test. Furthermore, this paper investigates the moderating role of equity incentives and institutional investors on the relationship between managerial myopia and green innovation. The results show that equity incentives and institutional investors both weaken the negative correlation between managerial myopia and green innovation.

This paper has several contributions. Firstly, this study combines the time-oriented theory in social psychology with the upper echelon theory in management and applies it to the field of enterprise green innovation. Secondly, this study contributes to upper echelons theory by investigating the effect of managerial myopia on firms’ green innovation and enriches the literature on the influence of managers’ characteristics on enterprises’ economic consequences. Thirdly, this study is an important supplement to the research on the driving factors of green innovation. Finally, this study contributes to the construction of managerial myopia indicators. Using text analysis and machine learning methods to construct managerial myopia indicators, this paper makes the measurement of manager myopia more direct and objective, solves the subjective bias and contextual problems of the questionnaire method, and provides an important reference for managerial myopia quantification.

The remainder of this article is organized as follows. I review the previous literature and propose research hypotheses in section “Literature Review and Research Hypotheses.” Section “Methodology” provides the data, the model specification, and the estimation method. Section “Empirical Results” provides the empirical results. Finally, I conclude my paper and discuss limitations and future works in section “Conclusion.”

## Literature Review and Hypotheses Development

### Driving Factors of Green Innovation

The environmental and organizational factors that influence green innovation have been widely explored. The main environmental factors that affect firm green innovation include coercive environmental regulations (Yuan and Xiang, 2018; Zhang et al., 2020), incentive policy including tax and credit incentives (Lanoie et al., 2011; Cao and Chen, 2019), market pressure from consumers, suppliers and competitors (Cao and Chen, 2019; Wang et al., 2021), institutional pressure (Zhou J. et al., 2021), green knowledge sharing among supply chain members (Song et al., 2020), and market demand (Horbach, 2008). The organizational factors that affect green innovation performance are mainly innovation capabilities and resources (Cuerva et al., 2014; Baeshen et al., 2021), sustainable human capital (Baeshen et al., 2021), green organizational identity (Xu et al., 2021), state ownership (Pan et al., 2020), and market orientation (Akhtar et al., 2021).

It is worth noting that besides organizational and environmental factors, there is another extremely important factor that will affect green innovation, which is, managers’ personal characteristics. Upper echelons theory suggests that executives’ experiences, values, cognitions, and personalities will affect firms’ strategy choices and outcomes (Hambrick and Mason, 1984; Hambrick, 2007). In other words, executives have bounded rationality and put a lot of their knowledge, cognition, and values into the enterprise’s strategic choice. As firm’s decision-maker, executives have the power to influence green innovation strategy. Although green innovation can save cost and increase firm’s sustainability, it has the characteristics of high expense and high risk of failure, thus how executives perceive green innovation is very important for enterprises’ engagement in green innovation. Recent studies have started to explore the role of executive characteristics on firm green innovation based on upper echelons theory. It has been investigated that managers’ academic experience (Zhao et al., 2021), CEO education (Zhou M. et al., 2021), CEO hubris (Arena et al., 2018), CEO religiosity (Iguchi et al., 2021), CEO political connection (Huang et al., 2021), and CEO hometown identity (Ren et al., 2021) can facilitate firms’ green innovation. However, one important characteristic, managerial myopia, has not been examined yet.

### Impact of Managerial Myopia on Green Innovations

Social psychologists believe that people have different time orientations, that is, people have different perceptions, concerns and insights into the past, present and future (Lewin, 1942). Temporal orientation is generally regarded as an innate and stable personal trait (Goldrich, 1967) and a subconscious process (Zimbardo and Boyd, 1999). Different temporal orientation determines how people choose and pursue social goals, thereby affecting people’s cognitive, emotional and behavioral motivations (Carstensen et al., 1999). Time orientation in management disciplines refers to manager’s subjective preference for the past, present and future in the process of strategic decision-making (Mosakowski and Earley, 2000; Bluedorn and Martin, 2008; Lumpkin and Brigham, 2011). Short-term orientation means that managers are subjectively focused on and value the present (Lin et al., 2019). Managers’ short-term oriented time cognition directly leads to managerial myopia. Instead of focusing on the future development of the enterprise, myopic managers are more inclined to focus on the benefits that can be satisfied immediately (Stein, 1989; Laverty, 1996).

Managerial myopia reflects the personal characteristics of managers’ perception of time, and managers’ cognition and characteristics will affect managers’ behavior and strategic choices, which in turn affect organizational behavior and results (Hambrick and Mason, 1984). Therefore, according to the theory of upper echelons theory, managerial myopia will affect the strategy and investment behavior of enterprises. Ridge et al. (2014) conduct content analysis of letters to shareholders to measure temporal myopia and find that temporal myopia creates a focus on the firm’s current strategy rather than long-term strategy. Due to greater emphasis on current performance, myopic managers prefer to maximize short-term financial performance at the expense of the long-term interests of the enterprise. Therefore, when making investment decisions, myopic managers are more inclined to choose projects with short-term and high returns (Narayanan, 1985; Stein, 1988; Holmstrom, 1999) rather than long-term and uncertain investments such as R & D. It has been proved that managerial myopia has led to a reduction in real investment and research and development (R&D) investment (Edmans et al., 2017; Kraft et al., 2018; Ladika and Sautner, 2020). Brochet et al. (2015) use text analysis of conference calls to capture managers’ subjective perception of time, their result show that short-term oriented managers are more likely to exhibit lower discretionary R&D expenditures. Green innovation requires continuous investment of a large amount of capital, with long cycles and high risks, which often leads to higher operating risks (Holmstrom, 1989). As a result, short-term-oriented managers will use their resources and power to influence the scale and direction of enterprises’ investment and reduce firms’ capital expenditures on green innovation, which will decrease firms’ green innovation output. Therefore, this article proposes the following hypothesis:

**Hypothesis 1:** Managerial myopia is negatively associated with enterprises’ green innovation.

### The Moderating Role of Equity Incentives and Institutional Investors

The core of equity incentive is to bind the long-term value of the company with the interests of managers, thereby restraining managers’ short-term behavior and reducing the principal-agent cost (Jensen and Meckling, 1976). Innovation usually has a long R & D cycle and a high risk of failure (Holmstrom, 1989), the traditional incentive method of linking performance and salary is not enough to effectively encourage the manager to invest in green innovation. The relatively long validity period of an equity incentive plan makes managers more committed to the long-term value of the enterprise, which will increase their support for R&D expenses on green innovation since green innovation is a long-term beneficial behavior for firm. It has been proved that CEO stock option incentive has a positive impact on R&D spending (Wu and Tu, 2007).

To conclude, by combining the interests of managers with the interests of the company for a long time and motivating managers to choose projects that are beneficial to company’s long-term development such as green innovation, equity incentives can alleviate the negative impact of manager’s short-sightedness on green innovation.

**Hypothesis 2:** Equity incentive weakens the negative relationship between managerial myopia and firms’ green innovation.

Compared with individual investors, institutional investors tend to have a large scale of funds, greater professional knowledge, and a stronger influence on corporate managers. They usually pursue value investing and have a stronger motivation to focus on and obtain long-term value information of the enterprise, rather than relying too much on short-term performance information (Stein, 1989). Therefore, institutional investors can effectively supervise managers to do things that are beneficial to the long-term development of the enterprise. In the long term, green innovation cannot only benefit firms by saving energy and cost, but also improve the ecological environment quality by reducing emissions (Sierzchula and Nemet, 2015; Dangelico et al., 2017), thus green innovation is a long-term beneficial behavior for enterprises. Therefore, when the shareholding ratio of institutional investors is higher, even if managers are myopic, their short-term behaviors on green innovation investment will be suppressed due to the supervision of institutional investors, and the negative correlation between managerial myopia and corporate green innovation will be weakened.

**Hypothesis 3:** Institutional investors weaken the negative relationship between managerial myopia and firms’ green innovation.

**FIGURE 1:**
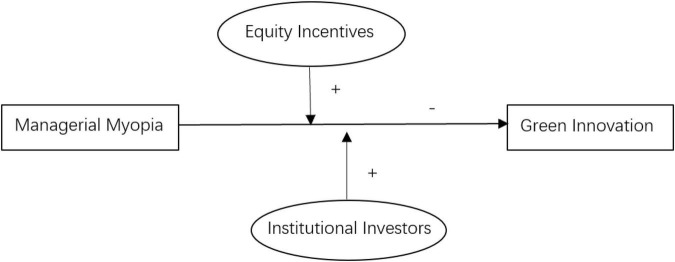
Theoretical model.

The proposed model of the study is shown in Figure 1.

## Methodology

### Sample and Data

This paper’s initial sample includes all Chinese listed firms in Shenzhen and Shanghai stock markets between 2009 and 2020. The final sample is selected according to the following criteria: First, we exclude “ST” firms because these firms are carried out special treatment by Shanghai and Shenzhen stock exchanges due to their abnormal financial conditions. Second, following previous studies, we remove financial firms due to their special industry attributes and operating characteristics. Finally, we delete observations within missing necessary data. Our final sample consists of 14,885 firm-year observations. All continuous variables are winsorized in the 1% quantile.

Green patent data is collected from the Chinese Research Data Services (CNRDS) database. MD & A text data comes from WinGo financial text data platform, which is the first financial text intelligent research platform in China, with professional Chinese financial text data analysis technology. Data of firm-level and manager characteristics are all from the China Stock Market and Accounting Research (CSMAR) database.

### Variable Definitions

#### Dependent Variable

Following previous research on green innovation (Huang et al., 2021; Ren et al., 2021; Zhou M. et al., 2021), this paper adopts the number of applications for green patents to measure the firms’ green innovation (Green_inno). In the robustness check, the number of applications for green inventions patents (IGpatent) and the number of applications for green utility patents (UGpatent) are alternative measure of green innovation.

#### Independent Variable

MD & A is managers’ review of the company’s operating conditions and the description of the next year’s business plan and the opportunities, challenges, and various risks faced by the company’s future development. Its text content can reflect the personal trait of managers. Taking the MD & A in the annual reports of Chinese A-share listed companies as the object, this paper determines the Chinese “short-term oriented” word set through text analysis (Li, 2010) and machine learning methods (Mikolov et al., 2013), and then use dictionary method (Loughran and McDonald, 2016) to construct the managerial myopia index. Specifically, the construction process of managerial myopia indicator is as follows:

1. Designate seed words. Drawing on the English “short-term oriented” word set (Brochet et al., 2015), through reading 200 MD & A corpora artificially, this paper identifies 10 seed words referring to the short-term orientation in Chinese MD & A. These 10 Chinese words translated into English are: “day(-s or daily),” “month(-s or -ly),” “year(-s or -ly),” “as soon as possible,” “immediately,” “at once,” “chance (opportunity),” “occasion,” “pressure,” “test.”
2. Expand similar words. This paper uses word2vec machine learning technology (Mikolov et al., 2013) to expand the similar words of “short-term oriented” seed words. The Word2Vec machine learning technique proposed by Mikolov et al. (2013) is a milestone achievement in this field in recent years. The essence of Word2Vec is based on the neural network Word Embedding method, which expresses words into multi-dimensional vectors according to contextual semantic information, and obtains the semantic similarity between words by calculating the similarity between vectors. This step forms 33 short-term Chinese words.
3. Calculate the managerial myopia index. Based on the dictionary method (Loughran and McDonald, 2016), this paper calculates the proportion of the total word frequency of 43 “short-term oriented” words in the total word frequency of MD & A and multiplies it by 100 to obtain the managerial myopia index. The larger the index value, the more myopic the manager is.

#### Moderating Variables

##### Equity Incentive

Equity incentive is measured by the ratio of the number of shares held by managers to the firm’s total shares.

##### Institutional Investor Shareholding

Institutional investor shareholding is measured by the ratio of the number of shares held by institutions to the firm’s total shares.

#### Control Variables

Following existing studies (Huang et al., 2021; Ren et al., 2021; Zhao et al., 2021; Zhou M. et al., 2021), the control variables in this paper include managerial background characteristics, firm-level characteristics, and corporate governance characteristics. Managerial background characteristics include gender, age, education, and tenure. Firm-level characteristics include the firm’s size, age, leverage, ROA, growth rate, R&D expense, and equity nature. As for corporate governance characteristics, this paper controls board independence and the largest shareholder’s shares proportion. The measurement methods of all variables are shown in Table 1.

**TABLE 1:** Variable definitions.

Variables	Definitions
Green_inno	The number of applications for green patents.
Myopia	(The total word frequency of “short-term oriented” words/the total word frequency of MD & A)* 100.
Degree	If CEO’s education background is high school graduate or below, then Degree equals 1, 2 for college graduate, 3 for undergraduate, 4 for master, 5 for doctorate
CEO_age	CEOs’ age.
Tenure	The number of years since the CEO has occupied the CEO position.
Gender	If CEO is female, then it equals 1 and 0 otherwise.
Firm_age	The number of years since the firm was established.
Size	The natural logarithm of total assets at the end of the year
Lev	Total liabilities over total assets.
Roa	Net income over total assets.
Soe	If the firm is controlled by the government or its various entities, it equals 1 and 0 otherwise.
Boardind	The number of independent directors over the total number of board members.
Growth	The annual revenue growth rate of the firm.
R&D	The natural logarithm of the amount of R&D investment.
First	The largest shareholder shareholding over total shares.
Institu_share	Institutional shareholding over total shares.
Manage_share	Manager shareholding over total shares.

### Empirical Models

To test Hypothesis 1, we use Ordinary Least Squares (OLS) to estimate the following models based on previous studies (Ren et al., 2021; Zhou M. et al., 2021):


(1) Green_Inno=itβ+0βMyopia1+itControl+Year+Industry+ε,it

where, i denotes firms and t represents time. Green_Inno represents firms’ green innovation and is measured in the number of green patent applications. Myopia is the independent variable that represents managerial myopia. Control is a set of control variables including all manager-level and firm-level characteristics. Year and Industry represent the time and industry fixed effects, respectively. To control the influence of the heteroscedasticity of the error term and the related problems of time series on the standard error of the estimated coefficient, this paper adopts the robust standard error and clusters the errors at the firm level. If the coefficient on Myopia is negatively significant, then hypothesis 1 is supported.

To test hypothesis 2 and 3, this paper divides the samples into two groups according to managerial shareholding ratio and institutional shareholding ratio, and conduct grouping regression for Model 1.

## Empirical Results

### Descriptive Statistics and Correlation Analysis

Table 2 provides the descriptive statistics. The mean of green innovation (Green_Inno) is 4.296, and the standard deviation is 7.780. The mean, median, and standard deviation of managerial myopia (Myopia) are, respectively, 0.077, 0.063, and 0.064. Summary statistics for CEO characteristics are as follows: the CEOs in the full sample are almost exclusively male, female only occupies 6.3%; they are on average 49 years old and their average tenure is 4 years; the mean of degree is 3.688, which is between bachelor’s degree and master’s degree. The descriptive statistical results of other control variables are basically consistent with the existing studies.

**TABLE 2:** Descriptive statistics.

Variables	Mean	Min	p50	Max	SD	N
Green_Inno	4.296	0.000	0.000	28.000	7.780	14,885
Myopia	0.077	0.000	0.063	0.255	0.064	14,885
CEO_age	49.530	38.000	50.000	59.000	5.795	14,885
Degree	3.688	1.000	4.000	7.000	1.311	14,885
Tenure	4.113	0.250	3.583	9.917	2.928	14,885
Gender	0.063	0.000	0.000	1.000	0.243	14,885
Firm_age	15.950	7.000	16.000	26.000	5.212	14,885
Size	22.000	20.210	21.840	24.560	1.121	14,885
Lev	0.394	0.101	0.383	0.793	0.191	14,885
Roa	0.048	–0.048	0.043	0.147	0.045	14,885
Growth	0.165	–0.284	0.125	0.846	0.261	14,885
SOE	0.279	0.000	0.000	1.000	0.449	14,885
Boardind	0.372	0.333	0.333	0.462	0.045	14,885
R&D	17.740	14.990	17.750	20.200	1.290	14,885
First	33.820	13.750	31.950	63.000	13.640	14,885

Table 3 displays the Pearson correlation matrices. The Pearson correlation coefficient of Myopia and Green_Inno is −0.05, which is significant at 1% level, showing that managerial myopia is significantly negatively related to green innovation, which is consistent with H1.

**TABLE 3:** The Pearson correlation matrices.

	Green_Inno	Myopia	CEO_age	Degree	Tenure	Gender	Firm_age	Size
Green_Inno	1							
Myopia	−0.050***	1						
CEO_age	0.056***	0.018**	1					
Degree	0.078***	−0.025***	−0.079***	1				
Tenure	0.026***	0.006	0.218***	–0.010	1			
Gender	−0.035***	–0.010	−0.039***	–0.012	–0.001	1		
Firm_age	0.082***	0.023***	0.144***	−0.015*	0.037***	0.021**	1	
Size	0.408***	0.052***	0.124***	0.079***	0.059***	−0.042***	0.203***	1
Lev	0.248***	0.102***	0.041***	0.023***	–0.008	−0.043***	0.143***	0.541***
Roa	−0.049***	−0.087***	−0.014*	0.0100	0.037***	0.023***	−0.074***	−0.065***
Growth	–0.010	−0.104***	−0.055***	0.040***	−0.026***	0.001	−0.077***	0.015*
SOE	0.142***	0.147***	0.106***	0.038***	−0.086***	−0.073***	0.167***	0.364***
Boardind	0.001	−0.047***	0.013	0.026***	0.005	0.066***	−0.024***	−0.028***
R&D	0.408***	−0.087***	0.063***	0.098***	0.091***	−0.052***	0.068***	0.534***
First	0.011	0.052***	0.028***	−0.021**	−0.083***	0.023***	−0.092***	0.133***

	**Lev**	**Roa**	**Growth**	**SOE**	**Boardind**	**R&D**	**First**	

Lev	1							
Roa	−0.389***	1						
Growth	0.010	0.314***	1					
SOE	0.309***	−0.157***	−0.103***	1				
Boardind	−0.032***	–0.008	0.003	−0.098***	1			
R&D	0.183***	0.123***	0.058***	0.083***	0.005	1		
First	0.060***	0.069***	−0.034***	0.198***	0.057***	0.011	1	

*, **, *** indicate significance at the 10, 5, and 1% levels, respectively.

### The Impact of Managerial Myopia on Firms’ Green Innovation

The regression analysis results of managerial myopia on firms’ green innovation are shown in Table 4. Column 1 displays the single effect of managerial myopia on firms’ green innovation. Column 2 shows the impact of all the control variables on green innovation. As shown in column 3 of Table 4, the coefficient of Myopia is significantly negatively correlated with Green_Inno (β = −2.241, *p* < 0.01). In terms of economic implications, on average, every one standard deviation increase in managerial myopia makes enterprise green innovation decrease by about 1.9% (=−2.241 × 0.064/7.780) standard deviation. It can be seen that managerial myopia is significantly negatively associated with enterprises’ green innovation both statistically and economically. Therefore, H1 is supported. This result shows that managers’ personal characteristics do affect enterprises’ strategy choices and economic outcomes, which supports the upper echelons theory (Hambrick and Mason, 1984; Hambrick, 2007), and is consistent with the result of literature studying the effect of managers’ characteristics on firms’ green innovation based on the upper echelons theory (Arena et al., 2018; Iguchi et al., 2021; Ren et al., 2021; Zhao et al., 2021; Zhou M. et al., 2021). As firm’s decision-maker, managers have the power to influence green innovation strategy. When making investment decisions, myopic managers are more inclined to choose short-term projects with high returns. Green innovation requires continuous investment of a large amount of capital, with long cycles and high risks. As a result, myopic managers will use their power to influence enterprises’ investment and reduce firm’s capital expenditures on green innovation, which will cause a decrease in firms’ green innovation output.

**TABLE 4:** The impact of managerial myopia on green innovation.

	(1)	(2)	(3)
Variables	Green_Inno	Green_Inno	Green_Inno
Myopia	−5.088***		−2.241***
	(−6.12)		(−2.93)
CEO_age		0.012	0.009
		(1.06)	(0.85)
Degree		0.083*	0.089*
		(1.78)	(1.92)
Tenure		−0.012	−0.002
		(−0.63)	(−0.11)
Gender		0.283	0.309
		(1.10)	(1.22)
Firm_age		0.122***	0.118***
		(7.68)	(7.56)
Size		1.759***	1.879***
		(18.49)	(19.02)
Lev		1.478***	1.022**
		(3.42)	(2.36)
Roa		−5.771***	−5.190***
		(−4.18)	(−3.78)
Growth		−1.458***	−1.514***
		(−8.23)	(−8.54)
SOE		0.267	0.356*
		(1.21)	(1.65)
Boardind		2.540*	2.284*
		(1.82)	(1.66)
R&D		1.339***	1.233***
		(20.70)	(17.78)
First		−0.023***	−0.023***
		(−3.84)	(−3.88)
Constant	4.358***	−61.378***	−61.664***
	(32.59)	(−34.81)	(−31.13)
Observations	14,885	14,885	14,885
R-squared	0.002	0.214	0.268
Year FE	Yes	Yes	Yes
Industry FE	Yes	Yes	Yes

All equations are estimated by OLS. The t-statistics in parentheses are calculated based on standard errors clustered at the firm level. *, **, *** indicate significance at the 10, 5, and 1% levels, respectively.

### The Moderation Effect of Equity Incentive

Taking the median of managerial shareholding as the boundary, this paper divides the samples into a high managerial shareholding group and a low managerial shareholding group, and performs group regression for model (1). Column 1 of Table 5 is the regression result of the sample group with low managerial shareholding, and column 2 is the regression result of the sample group with high managerial shareholding. As shown in column 2, for companies with a high managerial shareholding ratio, the impact of managerial myopia on the company’s green innovation disappears; while for companies with low managerial shareholding levels, as shown in column 1, manager myopia still has a significant negative impact on corporate green innovation (β = −1.849, *p* < 0.01). This result indicates that equity incentives for managers can alleviate the negative impact of managerial myopia on green innovation, thus H2 is supported. Equity incentives combine the interests of managers with the interests of the company for a long time and enable managers to attach importance to green innovation from the perspective of corporate long-term development, which can alleviate the negative impact of managerial myopia on green innovation.

**TABLE 5:** The moderating role of equity incentive and institutional investor.

	(1)	(2)	(3)	(4)

	Managerial shareholding		Institutional shareholding	

	Low	High	Low	High
Variables	Green_Inno	Green_Inno	Green_Inno	Green_Inno
Myopia	−1.849***	−1.156	−2.376***	−1.871
	(−3.76)	(−1.04)	(−3.21)	(−1.21)
CEO_age	0.015	−0.029*	−0.006	0.026*
	(1.09)	(−1.69)	(−0.34)	(1.91)
Degree	0.162***	0.009	0.046	0.112**
	(2.86)	(0.13)	(0.61)	(2.00)
Tenure	−0.010	0.027	−0.006	0.024
	(−0.38)	(0.95)	(−0.22)	(0.88)
Gender	0.074	0.741*	0.816**	−0.392
	(0.23)	(1.87)	(2.04)	(−1.20)
Firm_age	0.078***	−0.085***	0.189***	0.046**
	(3.91)	(−2.85)	(7.92)	(2.34)
Size	1.657***	1.833***	1.941***	1.789***
	(11.60)	(12.82)	(13.39)	(12.68)
Lev	1.107**	1.828***	1.174*	1.176**
	(1.98)	(2.71)	(1.79)	(2.04)
Roa	−6.387***	−3.173	−4.886**	−6.318***
	(−3.63)	(−1.46)	(−2.33)	(−3.52)
Growth	−1.320***	−1.566***	−1.493***	−1.368***
	(−5.64)	(−5.76)	(−5.58)	(−5.80)
SOE	0.177	0.832***	0.022	0.981***
	(0.56)	(2.79)	(0.07)	(2.62)
Boardind	−1.265	3.775*	5.707***	−0.393
	(−0.73)	(1.78)	(2.66)	(−0.22)
R&D	1.281***	1.064***	1.275***	1.154***
	(12.73)	(10.95)	(13.82)	(10.40)
First	−0.026***	−0.013	−0.022**	−0.028***
	(−2.86)	(−1.48)	(−2.53)	(−3.55)
Constant	−54.927***	−56.978***	−66.143***	−56.459***
	(−19.46)	(−18.85)	(−22.21)	(−20.99)
Observations	7,434	7,451	7,310	7,575
R−squared	0.233	0.301	0.278	0.237
Year FE	YES	YES	YES	YES
Industry FE	YES	YES	YES	YES

All equations are estimated by OLS. The t−statistics in parentheses are calculated based on standard errors clustered at the firm level. *, **, *** indicate significance at the 10, 5, and 1% levels, respectively.

### The Moderation Effect of Institutional Investor

This paper divides the samples into a high institutional shareholding group and a low institutional shareholding group according to the median of institutional shareholding. Then, we perform group regression for model (1), and the results are shown in Table 5. Column 3 of Table 5 is the regression result of the sample group with low institutional shareholding, and column 4 is the regression result of the sample group with high institutional shareholding. As shown in column 3 and column 4 of Table 5, for companies with low institutional shareholding levels, managerial myopia has a significant negative impact on corporate green innovation (β = −2.376, *p* < 0.01); while for companies with a high proportion of institutional investors shareholding, the impact of managerial myopia on the company’s green innovation disappears. This indicates that the higher institutional shareholding ratio strengthens investors’ supervision on companies, and the impact of managerial myopia on green innovation has been suppressed, thus H3 is supported. Institutional investors usually pursue value investing and have a stronger motivation to focus on and obtain long-term value of the enterprise. In the long term, green innovation can benefit firms by reducing waste and saving cost. Therefore, when the shareholding ratio of institutional investors is higher, even if managers are myopic, their negative effect on green innovation will be suppressed.

### Robustness Tests

In this section, we conduct several robustness tests. Firstly, to clarify that our previous findings are not driven by certain sample interval or variable measurements, we re-estimate our basic regression by altering sample interval and using an alternative green innovation indicator. Secondly, considering that many listed companies’ number of green patents applications is zero, the dependent variable (Green_Inno) naturally has a left-hand critical value (Zhou M. et al., 2021). In this case, the traditional ordinary least squares (OLS) estimation may be biased. Tobit model is a kind of model that the dependent variable is distributed continuously on the positive value, but it contains many observations with a positive probability value of 0. Therefore, we use Tobit regression to estimate Model (1) to exclude our concern. Thirdly, in order to more effectively control the omitting variables that do not change over time at the firm level, we use firm fixed effects model to re-test the main hypothesis. Finally, to offset the concerns about the endogenous selection criteria on hiring CEOs, we perform a 2SLS with an instrumental variable to exclude such a concern. The specific analysis is as follows.

#### Alternative Sample Interval

We extend the sample period from 2009–2020 to 2001–2020 and conduct similar test with model (1), regression results are shown in column 1 of Table 6. The coefficient of Myopia is significantly negative (β = −2.116, *p* < 0.01) when the sample period is altered, indicating that the negative effect of managerial myopia on firm green innovation is robust.

**TABLE 6:** Test of robustness.

	(1)	(2)	(3)	(4)	(5)	(6)

Variables	Green_Inno	IGpatent	UGpatent	Tobit model	Green_Inno	2SLS
Myopia	−2.116***	−1.493***	−0.512***	−10.553***	−2.002***	−10.895***
	(−5.36)	(−2.99)	(−3.12)	(−6.31)	(−2.61)	(−3.35)
CEO_age	0.008	−0.000	0.001	−0.002	0.011	0.014
	(1.23)	(−0.03)	(0.18)	(−0.09)	(1.06)	(1.39)
Degree	0.074**	0.041	0.051*	0.359***	0.082*	0.183***
	(2.48)	(1.38)	(1.90)	(4.56)	(1.75)	(4.21)
Tenure	−0.000	0.023*	−0.002	0.058	−0.012	−0.031
	(−0.01)	(1.90)	(−0.20)	(1.64)	(−0.60)	(−1.53)
Gender	0.008	0.185	−0.032	−0.428	0.281	−0.184
	(0.06)	(1.12)	(−0.22)	(−0.98)	(1.09)	(−0.79)
Firm_age	0.084***	0.059***	0.046***	−0.065***	0.119***	−0.001
	(9.16)	(5.99)	(5.21)	(−2.84)	(7.47)	(−0.06)
Size	1.640***	0.915***	0.792***	2.840***	1.757***	1.538***
	(34.08)	(14.43)	(13.84)	(19.09)	(18.47)	(20.57)
Lev	0.794***	0.103	0.887***	3.496***	1.530***	2.734***
	(3.26)	(0.37)	(3.51)	(4.70)	(3.54)	(6.84)
Roa	−3.199***	−3.426***	−1.890**	−5.845**	−5.760***	−6.013***
	(−4.09)	(−3.93)	(−2.37)	(−2.11)	(−4.18)	(−4.06)
Growth	−0.880***	−0.782***	−0.723***	−1.685***	−1.486***	−0.817***
	(−8.71)	(−6.88)	(−6.91)	(−3.93)	(−8.37)	(−3.38)
SOE	0.159	0.412***	0.074	1.155***	0.302	0.426***
	(1.41)	(3.12)	(0.63)	(4.42)	(1.37)	(2.81)
Boardind	−0.741	1.091	1.353*	−1.459	2.525*	1.225
	(−0.91)	(1.25)	(1.71)	(−0.64)	(1.81)	(0.96)
R&D	0.088***	0.730***	0.607***	2.912***	1.331***	1.634***
	(17.29)	(15.64)	(14.37)	(24.65)	(20.55)	(28.67)
First	−0.019***	−0.009**	−0.010***	−0.044***	−0.022***	−0.014***
	(−5.91)	(−2.50)	(−3.17)	(−5.65)	(−3.81)	(−3.28)
Constant	−35.448***	−31.613***	−28.029***	−115.793***	−60.991***	−59.648***
	(−32.31)	(−25.31)	(−25.00)	(−39.32)	(−34.49)	(−39.85)
Observations	23,697	11,623	11,623	14,885	14,885	14,885
R−squared	0.231	0.240	0.262		0.216	0.230
Pseudo R2				0.080		
First−stage F value						144.81
Firm FE	No	No	No	No	Yes	No
Year FE	Yes	Yes	Yes	Yes	Yes	Yes
Industry FE	Yes	Yes	Yes	Yes	No	Yes

The t-statistics in parentheses are calculated based on standard errors clustered at the firm level. *, **, *** indicate significance at the 10, 5, and 1% levels, respectively.

#### Alternative Dependent Variable

Green patents can be divided into two types: green invention patents and green utility patents. Green invention patent refers to a new technical solution for a product, a method or an improvement thereof, and utility patent refers to a new technical solution suitable for practical use proposed for the shape or structure of products. We use the number of applications for green inventions patents (IGpatent) and the number of applications for green utility patents (UGpatent) to measure firm green innovation separately and run similar regression with model (1), regression results are shown in column 2 and column 3 of Table 6, respectively. The coefficient of Myopia is still significantly negative when the dependent variable is IGpatent (β = −1.493, *p* < 0.01) or UGpatent (β = −0.512, *p* < 0.01), indicating that the negative effect of managerial myopia on firm green innovation still exists.

#### Alternative Model Regression

Considering that many listed companies’ number of green patents application is zero, the OLS estimation may be biased. Tobit model is a kind of model that the dependent variable is distributed continuously on the positive value, but it contains many observations with a positive probability value of 0. Therefore, we use Tobit regression to examine the impact of manager myopia on firm green innovation. As shown in column 4 of Table 6, the negative effect of managerial myopia on firm green innovation still exists (β = −10.553, *p* < 0.01).

#### Firm Fixed Effect Model

To offset the concerns about the endogenous problems caused by omitting variables, we control firm fixed effect in model (1) to more effectively control the omitting variables that do not change over time at the firm level. As shown in column 5 of Table 6, managerial myopia is still significantly negative correlated with green innovation (β = −2.002, *p* < 0.01).

#### 2SLS Regression

Enterprises may have selection criteria when recruiting managers, especially the growing enterprises that require the leadership of long-term orientation rather than myopia. we use instrumental variable and 2SLS regression to exclude such a concern. The average manager myopia level of other firms within the same industry and region is used to be an instrumental variable for Myopia. The first stage regresses Myopia with instrumental variables and other control variables to obtain the fitting value of Myopia. The second stage uses the fitting value of the first stage to run the least square regression of model (1), the result is shown in Column 6 of Table 6. The previous findings of managerial myopia and firm green innovation remain significantly negative (β = −10.895, *p* < 0.01).

## Conclusion, Implications, and Future Works

### Conclusion

Drawn on time-oriented theory in social psychology and upper echelon theory in management, this paper aims to examine the effect of managerial myopia on firm green innovation. Taking the MD & A in the annual reports of Chinese listed companies from 2009 to 2020 as the object, this paper constructs the managerial myopia index by text analysis and machine learning methods, then explores the effect of managerial myopia on firm green innovation and the moderating role of equity incentive and institutional investors. The research results show that: (1) managerial myopia is significantly negatively associated with enterprises’ green innovation. Every one standard deviation increase in managerial myopia makes enterprise green innovation decrease by about 1.9% standard deviation. (2) Equity incentive weakens the negative effect of managerial myopia on green innovation. (3) Institutional investors weaken the negative correlation between managerial myopia and green innovation. The results remain significant after a series of robustness checks including alternative sample interval, alternative dependent variable measures, alternative model regression, and endogenous tests.

### Theoretical Implications

This paper provides several theoretical contributions. Firstly, this paper combines the time-oriented theory in social psychology (Stein, 1989; Laverty, 1996) with the upper echelon theory (Hambrick and Mason, 1984; Hambrick, 2007), applies it to the field of enterprise green innovation, and expands the research on the behavioral economic consequences of managers’ myopia. Secondly, this study contributes to upper echelons theory (Hambrick and Mason, 1984; Hambrick, 2007) by investigating the effect of managerial myopia on firms’ green innovation and enriches the literature on the influence of managers’ personal characteristics on enterprises’ economic consequences. Thirdly, this study is an important supplement to the research on the driving factors of green innovation. Recent studies have started to explore the role of executive characteristics on firm green innovation, such as managers’ academic experience (Zhao et al., 2021), CEO education (Zhou M. et al., 2021), CEO hubris (Arena et al., 2018), CEO religiosity (Iguchi et al., 2021), CEO political connection (Huang et al., 2021), and CEO hometown identity (Ren et al., 2021). However, one important characteristic, managerial myopia, has not been examined yet. This study not only explores the influence of executives’ personal trait on green innovation but also investigates the moderating role played by internal incentives and external supervision. Finally, this study contributes to the construction of managerial myopia indicators. Using text analysis and machine learning methods to construct managerial myopia indicators, this paper makes the measurement of manager myopia more direct and objective, solves the subjective bias and contextual problems of the questionnaire method, and provides an important reference for managerial myopia quantification.

### Practical Implications

This paper also provides important implications for promoting green innovation and sustainable development for enterprises. First, the research results of this study show that the management’s long-term development cognition is the key to promoting the green innovation of the enterprise and achieving sustainable development. Second, the research results of this study provide instructional significance for the appointment of management talents in enterprises. When recruiting and training senior managers, companies should not only pay attention to their age, educational background, work experience, and other demographic characteristics but also focus on managers’ temporal cognition. Third, this paper confirms that internal incentives and external supervision can inhibit the negative effect of managerial myopia on green innovation. To promote the sustainable and healthy development of enterprises, firms should further improve the equity incentive mechanism and tie the interests of managers with the long-term interests of the enterprise; institutional investors should give full play to the role of external supervision on managers.

### Limitations and Future Works

Although this study attempts to clarify the impact of manager myopia on enterprise green innovation, due to the complexity of the research mechanism and the limitations of research methods, this study still has some deficiencies that need to be further deepened in future research. Firstly, this paper only takes the management discussion and analysis in the annual report as the text analysis object when constructing manager myopia index. In fact, many other corporate reports can also reflect the characteristics of management. Future research could expand the research scope, such as text analysis of social responsibility reports and internal control reports. Secondly, although this paper alleviates the endogenous problems to some extent, there may be more appropriate methods to deal with the concerns. Future research should continue to find more appropriate instrumental variables to deal with endogenous problems. Finally, the conclusions found in this study were only verified in the context of China, which may limit the generalizability of the findings. Future research could explore a wider range of research in the context of other countries to broader the theories.

## Data Availability Statement

The original data supporting the conclusions of this article will be made available by the author, without undue reservation.

## Author Contributions

The author confirms being the sole contributor of this work and has approved it for publication.

## Conflict of Interest

The author declares that the research was conducted in the absence of any commercial or financial relationships that could be construed as a potential conflict of interest.

## Publisher’s Note

All claims expressed in this article are solely those of the authors and do not necessarily represent those of their affiliated organizations, or those of the publisher, the editors and the reviewers. Any product that may be evaluated in this article, or claim that may be made by its manufacturer, is not guaranteed or endorsed by the publisher.

## Footnotes

1 Data sources: https://www.statista.com/statistics/239093/co2-emissions-in-china/
